# Diagnosing Pancreatic Adenocarcinoma With Contrast-Enhanced Ultrasonography: A Literature Review of Research in Europe and Asia

**DOI:** 10.7759/cureus.22080

**Published:** 2022-02-10

**Authors:** Aaiz Hussain, Derek S Weimer, Nisha Mani

**Affiliations:** 1 Medical School, Dr. Kiran C. Patel College of Allopathic Medicine, Nova Southeastern University, Davie, USA; 2 Department of Radiology, Aventura Hospital and Medical Center, Aventura, USA

**Keywords:** pancreas imaging, ultrasound imaging, contrast enhanced ct, contrast-enhanced ultrasound, ductal pancreatic adenocarcinoma

## Abstract

The National Cancer Institute names pancreatic cancer the 11^th^ most common type of cancer in the United States. However, even with a somewhat low prevalence, in 2017, the American Cancer Society reported pancreatic cancer as the fourth leading cause of cancer-related death. With a lack of symptomology and a broad range of risk factors, pancreatic cancer is frequently diagnosed in a later phase than many other types of cancers, thus resulting in higher metastasis along with a poorer prognosis. This highlights the need for early detection and diagnosis. Currently, abdominal ultrasound or contrast-enhanced CT imaging of the abdomen are standard of care. A new technology: contrast-enhanced ultrasound (CEUS), which employs contrast agents to act as acoustic enhancers for ultrasound, has FDA approval for use in hepatic and renal lesions, but not pancreatic. By examining seven individual studies from Europe and Asia, this review aims to examine the diagnostic value of CEUS to initially diagnose pancreatic adenocarcinomas, potentially followed by a biopsy to confirm, when compared against modalities currently used such as conventional ultrasound and CT imaging. CEUS would potentially be more accurate when compared to conventional ultrasound due to the addition of contrast, and when compared against CT and MRI, CEUS would be advantageous in its low cost, similar sensitivities, and specificities, limited renal toxicity, lack of ionizing radiation, short half-life, and its safe use in both adult and pediatric patients. Due to this, additional research is warranted for further FDA approval and future clinical implementation.

## Introduction and background

The National Cancer Institute states that pancreatic cancer represents 3.2% of all new cancer cases in the United States. This amounted to approximately 60,430 new cases in 2021 [1]. Despite pancreatic cancer being the 11^th^ most common type of cancer in the United States, the American Cancer Society reported pancreatic cancer as the fourth leading cause of cancer-related death in 2017, making this a major health concern within the United States [1-3]. In light of this, effective and timely diagnosis of pancreatic cancer at the earliest stage possible is critical to increased survival and quality of life. 

It is estimated that about 95% of malignant neoplasms affecting the pancreas are derived from acinar and/or ductal cells (i.e., exocrine cells) and typically resemble adenocarcinoma formation [4]. Thus, pancreatic cancer typically refers to an adenocarcinoma (i.e., a malignancy occurring within the glands of the organ) [4,5]. Patients who succumb to this disease typically remain asymptomatic, although jaundice is one of the primary symptoms indicative of this type of cancer, especially when the tumor is located in the head of the pancreas leading to biliary obstruction [6]. Other, more generalized, symptoms can exist as well, including steatorrhea, malabsorption, weight loss, abdominal discomfort, and abdominal bloating [3,4]. The risk factors for pancreatic cancer are generally broad in scope, including age, obesity, smoking, diabetes mellitus, and chronic pancreatitis [3,7]. Although familial history is relatively uncommon (about one in 10 cases), many hereditary genetic abnormalities, including BRCA2 gene mutation, can pose additional risks [8]. Despite the low prevalence of inherited pancreatic cancer, familial history and genetic susceptibility remain the main assessments of risk when considering patient enrollment within screening and early diagnosis programs [3,4]. 

With a lack of symptomology and a broad range of risk factors, it comes with no surprise that pancreatic cancer is diagnosed at a later stage than many other cancers, thus resulting in a higher prevalence of metastasis along with a poorer prognosis in an already challenging cancer to control [3,9]. From 2011 to 2017, 52% of diagnosed pancreatic cancer cases were labeled ‘distant’ or metastatic, with a five-year survival rate of about 3.0% [1]. Despite improvements within the field of oncology, pancreatic cancer persistently lacks prognostic improvements. The National Cancer Institute reported that since 1975, the death rate for this cancer has remained relatively stable [1]. Only 10% of pancreatic adenocarcinomas are resectable, with the final determination of resectability made intraoperatively. Imaging techniques used for the diagnosis and staging of pancreatic cancer include conventional ultrasound (US), endoscopic ultrasound (EUS), contrast-enhanced CT (CECT), magnetic resonance imaging (MRI), magnetic resonance cholangiopancreatography (MRCP), and endoscopic retrograde cholangiopancreatography (ERCP). Conventional US has an accuracy of 50% to 70%, while CT has a sensitivity of 90%, specificity of 87%, and diagnostic accuracy of 89%, and MRI has a sensitivity of 93%, specificity of 90%, and diagnostic accuracy of 87% [6,10]. This literature review aims to examine the diagnostic value of an emerging radiological modality: contrast-enhanced ultrasound (CEUS) for diagnosing pancreatic adenocarcinomas, potentially followed by a biopsy to confirm, compared to modalities currently used such as conventional US and CECT.

## Review

What is contrast-enhanced ultrasound (CEUS)?

There are various methods to diagnose pancreatic cancer. The simplest method is the use of biomarkers in serology testing, which is often conducted on patients who are considered ‘at-risk’ (i.e., family history and/or genetic susceptibility). Currently, there is only one serum biomarker with clinical relevance, carbohydrate antigen (CA) 19-9, a carbohydrate tumor-associated antigen. Though this may provide preliminary data in support of a suspected pancreatic cancer diagnosis, the specificity and sensitivity remain low [7]. Thus, serology lacks screening and diagnostic capabilities, leading to the importance of radiological imaging.

With pancreatic cancer, imaging plays an integral role in achieving an early diagnosis. Although several modalities exist, we will be discussing the most common imaging technologies used in United States clinical practice. On suspicion of pancreatic malignancies (such as patients presenting with jaundice), non-invasive methods are considered first. The conventional US is considered usually appropriate due to its non-invasive and cost-effective features. Despite potential benefits, the accuracy and sensitivity depend on the operator’s experience, making the sensitivity of B-Mode US about 40% to 91% [9,11]. When considering the various pancreatic regions, conventional US has greater potential in diagnosing malignancies within the head but difficulty when evaluating the neck and tail regions. Lee et al. attributes this to the presence of posterior acoustic shadowing from gastrointestinal gas bubbles. Without the use of contrast, the diagnosis becomes even more difficult due to the inability in deciphering between focal lesions and other diseases such as chronic pancreatitis and/or neuroendocrine tumors [9]. With these limitations, a confirmatory diagnosis often requires further imaging with either CT or MRI.

The high contrast resolution of CECT allows the radiologist to visualize vascular involvement, a critical factor in determining resectability [9,12]. The superior tissue contrast of MRI allows for increased diagnostic accuracy and image quality [9]. These non-invasive modalities greatly assist in the formation of a differential diagnosis and appropriate staging if a diagnosis of cancer is certain. However, due to both their high cost, the potential radiation exposure from CECT, and the inconvenience of the MRI, these modalities are not ideal for all patient populations [12]. EUS-Guided Needle Biopsy Aspiration and ERCP are more invasive methods, but they are important tools to differentiate pancreatic lesions and obtain tissue sampling, ensuring accurate diagnosis. However, they do pose risks of sedation, bleeding, and organ damage and cannot always determine the extent of the tumor, which is necessary for determining resectability [3].

CEUS, on the other hand, has screening and diagnostic potential while also considering economical, convenience, and safety considerations. CEUS is an innovative approach to radiological imaging with excellent diagnostic capabilities that provides a cross-sectional, detailed image of various tissues within the body utilizing a blood-pool microbubble ultrasound contrasting agent (UCA), with high contrast and spatial resolution, and real-time tumor enhancement using low acoustic US pressure [11,13-15].

**Figure 1:**
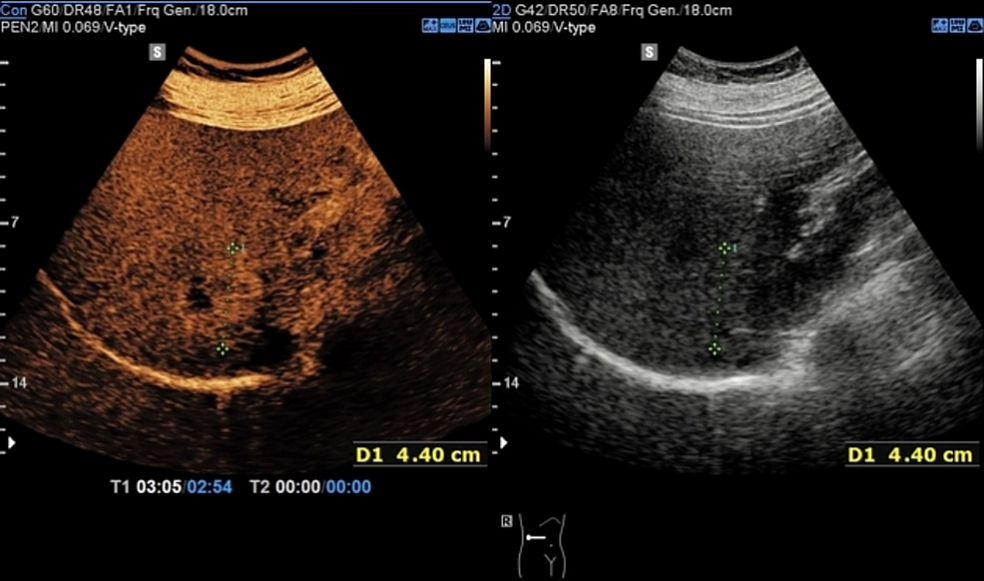
The liver lesion is barely seen on conventional B-mode US (on the right) compared to early arterial hyperenhancement (in the early post contrast phase) seen with CEUS using IV SonoVue (on the left). CEUS: contrast-enhanced ultrasound Image from Radiopaedia [18].

As we begin this review, it is important to understand the context and current applications of CEUS. CEUS has been in clinical use for pancreatic lesions in Asia and Europe for many years; however, the lack of Food & Drug Administration approval for this purpose has been limited in both clinical implementation and research in the United States. Currently, there are three second-generation UCAs with FDA approval for other uses: Lumason (i.e., SonoVue outside of the US; sulfur hexafluoride lipid-type A microspheres), Definity (Perflutren Lipid Microspheres), and Optison (Perflutren Protein-Type A) [16]. The secondary-contrast agents consist of microbubbles with a core of fluorinated gas, such as hexafluoride (Lumason; SonoVue) or octafluoropropane (Definity), and an outer superficial phospholipid layer allowing them to retain their shape when traveling through the body [17]. The contrast agents act as acoustic enhancers, creating harmonic responses that allow for increased signal in regions of interest while filtering background tissue signals. This allows for dynamic observation of the contrast-enhanced phases, ultimately providing a detailed image within seconds after injection of the agent [15,17]. When compared to other conventional imaging methods, no other modalities allow for continuous monitoring of enhancement during the dynamic phases [15]. Barr et al. highlight the current FDA approval for CEUS use. Lumason (SonoVue) is approved for use in detecting focal liver lesions (FLLs) in adult and pediatric patients, as well as use in the urinary tract for suspected or known vesicoureteral reflux in pediatric patients. Barr et al. performed a meta-analysis of 21 studies and found a pooled sensitivity of 88% and specificity of 81% for FLLs. [16]. The diagnostic value of CEUS in liver lesions can be visualized in Figure 1. Given this, clinical use of CEUS in the United States is limited when considering its potential diagnostic potential for pancreatic cancer.

**Figure 2:**
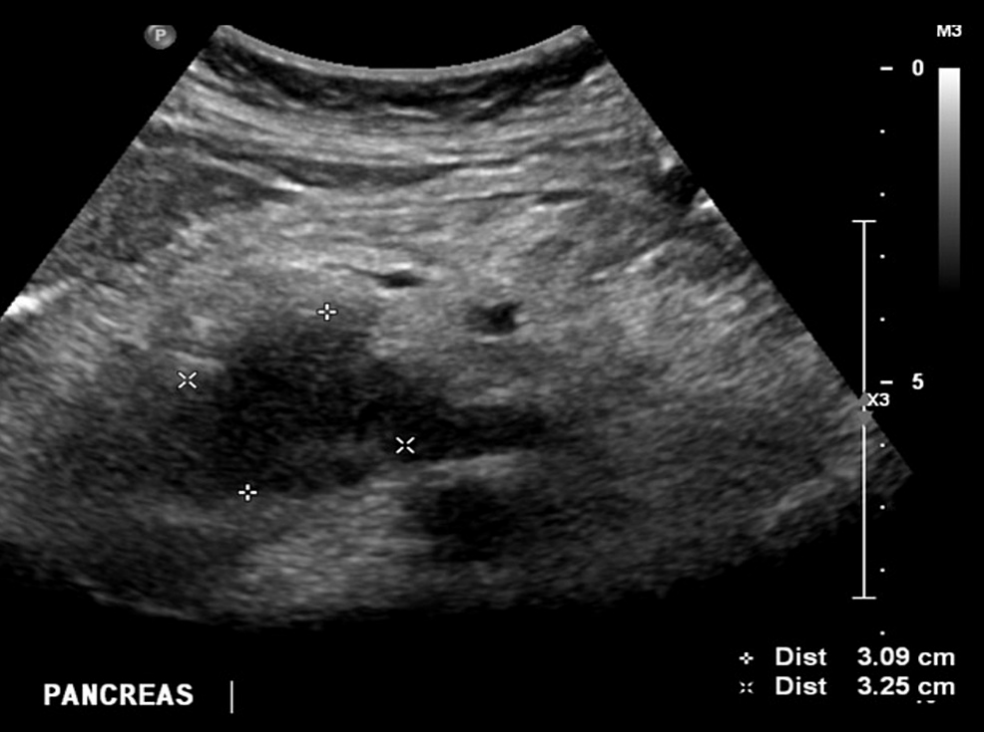
Longitudinal ultrasound images of the head/uncinate process of the pancreas shows hypoechogenic mass (due to decreased vascularity), pancreatic adenocarcinoma until proven otherwise. Image from Radiopaedia [21].

Several characteristics have been identified to differentiate various pancreatic carcinomas on ultrasound. Pancreatic adenocarcinoma, the most common carcinoma of the pancreas, presents as a solid hypoechoic mass (Figure 2) [19]. The second most frequent pancreatic solid neoplasms are neuroendocrine tumors which generally appear as hypervascular solid masses. Other possibilities on the differential when evaluating pancreatic lesions include metastasis from other organs, mass forming pancreatitis, in addition to cystic and solid pancreatic tumors, including serous and mucinous neoplasms [20]. While each entity has its imaging findings, this review will primarily focus on the efficacy of CEUS in the differentiation of pancreatic adenocarcinoma.

The literature

When reviewing the literature, our criteria was to evaluate research studies published in the timeframe of 2010 to 2021 that specifically looked at the effectiveness of CEUS in diagnosing and characterizing pancreatic adenocarcinoma with a hypo-enhancement pattern. Our criteria also included that the studies provided either diagnostic accuracy, sensitivity, specificity, or any combination of the three values specifically for pancreatic adenocarcinoma. We searched primarily for PubMed indexed articles. We, thus, excluded studies with accuracy or sensitivity/specificity values for other pancreatic lesions. Because of our criteria, we were able to include two meta-analyses that did provide a pooled specificity and sensitivity specifically for pancreatic adenocarcinoma. Each of the studies is further described below, but the main objective of this review is to stress that while CEUS is FDA approved for hepatic and renal lesions, the technology is very novel, with research in the United States for pancreatic lesions being non-existent. Five of the seven studies mentioned below are conducted in Asia, primarily in China and Japan, and two of the seven are performed in Europe, hence the title of our review. Thus, the United States is far from FDA approval and from becoming the standard of care for a disease as serious as pancreatic adenocarcinoma.

Tanaka et al. evaluated and compared the sensitivity of CEUS and CECT imaging for the characterization of small and early-stage pancreatic ductal adenocarcinoma by performing a prospective cohort study in Osaka, Japan. They looked at 6297 patients and used IV Sonazoid as their contrast agent. Of these, 231 patients were found with hypoechoic regions of 4 to 20 mm or less detected with ultrasound of patients in the sitting position after ingesting 350 mL of liquid. Of the 231 patients, 200 had further characterization of the lesions with CEUS and CECT. After CEUS and CECT, detailed examinations such as endoscopic ultrasound with fine-needle aspiration (EUS-FNA) and/or ERP-PJC were additionally performed to confirm the diagnosis. Of the 200 patients, 100 were found with invasive ductal adenocarcinoma, 76 had other benign lesions, and the remaining 24 had either non-invasive adenocarcinoma, neuroendocrine neoplasm, or metastatic adenocarcinoma from the lung. The study found a sensitivity for invasive ductal adenocarcinoma of CEUS to be 97% (95% CI: 93.6-100.4), which was significantly superior to that of CECT’s sensitivity of 77% (95% CI: 68.81-.85.39) with a p-value < 0.0001. However, overall accuracy was found to be similar, with CEUS representing 77% accuracy and CECT at 76% accuracy. Interestingly, the sensitivity of CEUS for characterizing small (<20mm) pancreatic adenocarcinomas (100%, 95% CI: 100-100) was significantly superior to that of CECT (76.7%, 95% CI: 63.6-89.9) with p=0.0016. Lastly, the sensitivity for characterizing even smaller (<10mm) adenocarcinomas was significantly superior for CEUS (100%, 95% CI: 100-100) than that of CECT (58.3%, 95% CI: 25.6-91.1) with p=0.0253 [22].

Grossjohann et al. performed a prospective study in Denmark to evaluate the viability of diagnosing pancreatic carcinoma in the head of the pancreas with CEUS. Their study evaluated CEUS against conventional ultrasound and CT imaging on 49 patients. 44 of these 49 patients had pancreatic head carcinoma. They were able to find a sensitivity of 86% for CEUS, 89% for conventional ultrasound, and 93% for CT imaging. Regarding accuracy, CEUS had an accuracy of 86%, conventional ultrasound had an accuracy of 82%, and CT imaging had an accuracy of 88%. They used SonoVue as their contrast agent. This study is interesting as it is the only study out of the ones reviewed where only the head of the pancreas is evaluated. It shows similar accuracy between the three modalities [23]. The benefit of CEUS, however, lies in potentially being able to evaluate other areas of the pancreas better than conventional ultrasound and being potentially cheaper than CT imaging with no radiation exposure.

Fan et al. performed a prospective cohort study in Beijing, China, to evaluate CEUS against conventional US and CT imaging. They enrolled 90 patients with solid pancreatic focal lesions. These lesions included: pancreatic adenocarcinoma, pancreatitis, neuroendocrine and pseudopapillary tumors. They used SonoVue as their CEUS contrast agent. The overall diagnostic accuracy of CEUS was found to be 83.33% against 44.44% of conventional ultrasound. Lastly, it found that for the diagnosis of pancreatic adenocarcinoma, the sensitivity was 91.7%, the specificity was 87.0%, and the accuracy was 88.9% [24].

Wang et al. performed a prospective cohort study in China by enrolling 210 patients with solid pancreatic lesions to investigate the value of CEUS against conventional US and CT imaging. They used SonoVue as their CEUS contrast agent. They created six diagnostic criteria to diagnose various lesions in the pancreas. To diagnose pancreatic carcinoma, they employed the criteria of hypo-enhancement or centripetal enhancement. When doing so, CEUS had an accuracy of 87.62% in diagnosing pancreatic carcinoma. They found that the diagnostic accuracy was significantly greater than that of conventional US but not significantly different than that of CT imaging [25]. However, once again, the benefit of CEUS, in this case, would be that the patients were not exposed to radiation, and CT imaging would be potentially cheaper.

Serra et al. completed a prospective study with 127 patients with undetermined pancreatic masses in Italy to investigate the diagnostic capabilities of CEUS in differentiating exocrine from neuroendocrine tumors. They used SonoVue as their contrast agent and injected about 2.4 mL using a 20-gauge catheter followed by a 10 mL flush of saline water. They found that diagnosing ductal adenocarcinoma with its hypo enhancing pattern on CEUS had a sensitivity of 91.3%, specificity of 85.3%, and diagnostic accuracy of 91.3%. They also found a positive predictive value of 94.7% and a negative predictive value of 90.6%, respectively [26].

Lin et al. completed a meta-analysis to evaluate pancreatic mass lesions. This study, conducted in China, included 18 eligible articles. Of these, 2,664 patients were included in the analysis. For secondary contrasting agents, SonoVue was administered in 12 studies, while Levovist was in six studies. For pancreatic adenocarcinoma, the study found a pooled estimated sensitivity of .90 (95% CI: .89-.92), with a pooled specificity of .88 (95% CI: .83-.89) [27].

Yamashita et al. performed a meta-analysis of studies that looked at the diagnosis of pancreatic carcinoma using CEUS. The study from Japan looked at nine studies with 983 patients. The studies used SonoVue or Sonazoid as their contrast agents. For diagnosing pancreatic carcinoma, they found a pooled specificity of 92% (95% CI: .89-.94) and specificity of 76% (95% CI: .71-.81) [28]. A summary table of all the studies (Table 1) has been included for easier reference.

**Table 1:** Summary of diagnostic accuracy, sensitivity and specificity of CEUS imaging in diagnosing pancreatic adenocarcinoma from research papers published after 2010 from Europe and Asia. CEUS: contrast-enhanced ultrasound

Study (Year Published)	Pancreatic Lesion studied	Diagnostic Accuracy	Sensitivity	Specificity
Tanaka et al. (2020) [22]	Invasive pancreatic adenocarcinoma	77%	97% (95% CI: 93.6-100.4)	-
Grossjohann et al. (2010) [23]	Pancreatic carcinoma of the head	86%	86%	-
Fan et al. (2013) [24]	Pancreatic adenocarcinoma	88.9%	91.7%	87.0%
Wang et al. (2021) [25]	Pancreatic adenocarcinoma	87.62%	-	-
Serra et al. (2013) [26]	Pancreatic adenocarcinoma	91.3%	91.3%	85.3%
Lin et al. (2016) [27]	Pancreatic adenocarcinoma	-	.90 (95% CI: .89-.92)	.88 (95% CI: .83-.89)
Yamashita et al. (2021)	Pancreatic carcinoma	-	92% (95% CI: .89-.94)	76% (95% CI: .71-.81)

Final considerations and future recommendations 

With sound methodologies, these studies outline that CEUS is not only sensitive and specific but also can be similarly accurate when compared to CT imaging. Currently, to the authors’ knowledge, there are no large-scale trials currently or previously conducted in the United States, while they have been performed in Europe and Asia. While we were limited in the number of available studies to review, there are other research papers available on this modality that are not as readily available in the United States due to language and specific journal barriers. Even though not much research has been conducted on this diagnostic technique in general, the authors of this review stress the many advantages of CEUS outlined in the current research. The first consideration is the safety profile. Compared to CT imaging, there is no radiation involved in CEUS. A CT abdomen with IV contrast exposes the patient to about 16 mSv of radiation [29]. Additionally, the contrast agents used for CEUS are generally excreted via exhalation from the lungs after the destruction of the microbubbles. This prevents nephrotoxicity when using second-generation contrasting agents, which is an important consideration when comparing against CT and MRI [30]. Since the contrast agents used in CEUS are still considered a “foreign body”, there have been reports of hypersensitivity reactions, but these are less common than from the use of iodine contrast agents in CT imaging and are similar to that of gadolinium used in MRI [31]. There are some contraindications for the use of SonoVue in patients with heart disease, such as recent acute coronary syndrome, right-to-left shunt, or ischemic cardiac disease. Further contraindications include severe pulmonary hypertension, uncontrolled hypertension, and acute respiratory distress syndrome (ARDS) [30]. An additional consideration is the economic benefits. While no true cost for CEUS is available for the diagnosis of pancreatic lesions/carcinoma, studies demonstrate that CEUS is more cost-effective than both CT imaging and MRI for FLLs, for example, in Europe [32].

Barr et al. note the sensitivity and specificity of CEUS in hepatic lesions, and they are not too dissimilar to the ones that the studies in Europe and Asia displayed when diagnosing pancreatic adenocarcinoma with CEUS [16]. However, CEUS use, even in hepatic and renal cases, is still very novel. Our recommendations would be for large-scale randomized controlled trials to be implemented, as CEUS provides an avenue to not only save cost when compared to CT imaging and MRI but also provide greater sensitivity than CT imaging, as Tanaka et al. identified, particularly with smaller lesions [22]. However, one major critique of the review was that the studies outside of Grossjohann et al. lacked information about the visibility of the tumors in the various locations of the pancreas (head, tail, and body) on CEUS imaging. The visibility of pancreatic tumors varies on regular ultrasound, yet the authors did not vigorously comment on the locations. Another critique is that Tanaka et al.'s studies do not delve into the sensitivity and specificity of CEUS when detecting pancreatic adenocarcinoma at varying sizes. A final critique is that the studies do not comment on the effectiveness of CEUS in staging pancreatic adenocarcinoma. Thus, FDA approval becomes plausible after and only after large-scale trials in the United States display comparable or greater diagnostic accuracy, sensitivity, and specificity than CT imaging in diagnosing pancreatic adenocarcinomas in each location of the pancreas at all sizes. If CEUS can do this, it can act as a cheaper screening tool, with CT imaging being done later to stage the tumor if it is found that CEUS cannot stage tumors. Overall, there would be cost-saving and reduced radiation exposure, as patients who do not have pancreatic adenocarcinoma would not have to get CT imaging done. Our prediction is if these trials were to happen, areas with decreased access to CT and MRI technology would benefit from clinical applications of CEUS. Thus, it is our recommendation for CEUS use to be further investigated for it can potentially become the next standard of care in the diagnosis of pancreatic adenocarcinoma.

## Conclusions

In conclusion, contrast-enhanced ultrasound is a novel diagnostic modality currently having FDA approval in the differentiation and diagnosis of hepatic and renal lesions in the United States. In the United States specifically, there exists a lack of controlled trials for the use of CEUS in diagnosing pancreatic cancer and other pancreatic lesions. By examining studies published from Europe and Asia, we were able to formulate a conclusion in determining that CEUS has vast potential in diagnosing pancreatic adenocarcinoma and differentiating it from other pancreatic lesions. By clinically implementing CEUS, it would not only reduce costs across the overall healthcare system when compared to CT and MRI imaging but will also reduce radiation exposure when compared to CT imaging. Through considering economical, safety, and convenience factors, we believe an increase in research is warranted for CEUS within the United States.
